# Pulmonary Alveolar Microlithiasis Coexisting With Rheumatic Heart Disease and Severe Pulmonary Hypertension: A Case Report

**DOI:** 10.7759/cureus.99674

**Published:** 2025-12-19

**Authors:** Aadithya Shyllesh H, Tehsim A Memon, Pranathi Guruswamy, Tufayl Ahmed M Shekha, Rutva Harish Fatnani

**Affiliations:** 1 Internal Medicine, Ramaiah Medical College Hospital, Ramaiah University of Applied Sciences, Bengaluru, IND; 2 Internal Medicine, Symbiosis Medical College for Women, Symbiosis University Hospital and Research Centre, Symbiosis International (Deemed) University, Pune, IND

**Keywords:** alveolar microliths, micro calcifications, pulmonary alveolar microlithiasis, pulmonary hypertension, rheumatic heart disease

## Abstract

Pulmonary Alveolar Microlithiasis (PAM) is a rare diffuse lung disease characterized by intra-alveolar calcium phosphate microlith accumulation. Its coexistence with rheumatic valvular heart disease with resultant severe pulmonary hypertension is exceedingly uncommon and poses unique diagnostic and management challenges. This case underscores the importance of maintaining a broad differential diagnosis, emphasizing that new or worsening respiratory symptoms in patients with established cardiac disease should not be automatically attributed to cardiac pathology alone.

We report the case of a 63-year-old gentleman with longstanding rheumatic heart disease (RHD) who presented with progressive dyspnoea. Clinical evaluation revealed features suggestive of severe pulmonary hypertension. Investigations confirmed PAM with characteristic “sandstorm” calcifications on chest imaging, supported by bronchoalveolar lavage demonstrating calcospherites and identification of an *SLC34A2 *gene mutation, alongside rheumatic mitral stenosis. The patient’s pulmonary hypertension was attributed to a combination of PAM and RHD.

This case illustrates a rare combination of PAM with RHD and severe pulmonary hypertension. It underlines the importance of considering dual pathology in patients with complex cardiopulmonary presentations. Early recognition of PAM, even in the presence of another disease like RHD, is crucial for appropriate management and prognostication. This report adds to the limited literature on such coexistence and highlights the need for multidisciplinary care in these patients.

## Introduction

Pulmonary alveolar microlithiasis (PAM) is an extremely rare lung disease characterized by the accumulation of innumerable microscopic calcium phosphate concretions (microliths) within the alveoli. Fewer than 1100 cases have been reported worldwide [1], and it typically follows an insidious course. Patients are often asymptomatic in early stages despite characteristic radiological findings - a phenomenon of clinico-radiological dissociation where chest imaging appears strikingly abnormal relative to mild clinical symptoms [1]. As the disease advances, however, patients may develop progressive dyspnoea, respiratory failure, and cor pulmonale. This phenomenon of clinico-radiological dissociation in pulmonary alveolar microlithiasis further complicates diagnostic evaluation in patients with pre-existing cardiac disease. In early and intermediate stages of PAM, striking radiological abnormalities may coexist with relatively mild or nonspecific respiratory symptoms. In patients already burdened with conditions such as rheumatic heart disease and heart failure, symptoms like exertional dyspnoea and fatigue are often readily attributed to the underlying cardiac pathology. This overlap can mask the presence of a concurrent pulmonary process, delaying consideration of alternative diagnoses and leading to underrecognition of PAM until advanced disease or incidental imaging findings prompt further investigation. A mutation in the *SLC34A2 *gene encoding a type IIb sodium-phosphate cotransporter in alveolar type II cells has been identified as the causative factor in a majority of cases [2]. This mutation leads to the formation and accumulation of calcium-phosphate microliths in alveolar spaces [2]

Rheumatic Heart Disease (RHD), on the other hand, is a post-inflammatory valvular disease following rheumatic fever, most commonly affecting the mitral valve and leading to stenosis or regurgitation. Chronic rheumatic mitral stenosis can result in pulmonary venous hypertension and reactive pulmonary arterial hypertension over time. While both PAM and RHD with secondary pulmonary hypertension (PH) are individually well-documented entities, their coexistence in a single patient is extraordinarily rare [3]. Only isolated instances could be identified in the literature, such as a 2007 report of a patient with PAM and rheumatic valvular disease [3]. The overlap of these conditions poses a unique diagnostic challenge - symptoms of breathlessness might be attributed to cardiac causes, potentially delaying the recognition of an underlying diffuse lung disease such as PAM. Our patient had severe pulmonary hypertension with significant hypoxemia, demonstrated definitive diagnostic confirmation through bronchoalveolar lavage showing calcospherites and molecular identification of an *SLC34A2 *mutation, and exhibited a clearly defined mixed pre- and post-capillary pulmonary hypertension physiology. These features add diagnostic certainty and highlight a more advanced cardiopulmonary disease burden compared to the previously reported case of PAM with rheumatic valvular disease described by Sampsonas et al. (2007) [3].

We hereby discuss a rare case of pulmonary alveolar microlithiasis coexisting with rheumatic heart disease and severe pulmonary hypertension. This unusual combination highlights the importance of maintaining a broad differential and adopting an interdisciplinary approach in complex cardiopulmonary presentations.

## Case presentation

A 63-year-old male, moderately built and poorly nourished, presented to a tertiary care hospital with a three-month history of progressively worsening dyspnea, particularly exacerbated by exertion, with New York Heart Association (NYHA) grade 3. This was accompanied by a productive cough yielding moderate amounts of yellowish sputum that was neither blood-stained nor foul-smelling, along with mild to moderate fever for four to five days. The patient had a significant past medical history of heart failure with preserved ejection fraction, rheumatic heart disease characterized by moderate mitral stenosis and mitral regurgitation with moderate tricuspid regurgitation, severe pulmonary arterial hypertension, and a left lower limb deep vein thrombosis.

Upon admission, the patient exhibited significant hypoxia, with an oxygen saturation of 83% on room air, which improved to 94% at 8 L/min using a non-rebreather mask. His respiratory rate was elevated at 27 breaths per minute, indicating tachypnea. Physical examination revealed bilateral pitting pedal edema and grade 3 digital clubbing. The BMI of the patient was 17.1. The respiratory examination indicated diminished air entry bilaterally across all lung fields, accompanied by occasional crepitations and a basal pleural rub.

**Figure 1:**
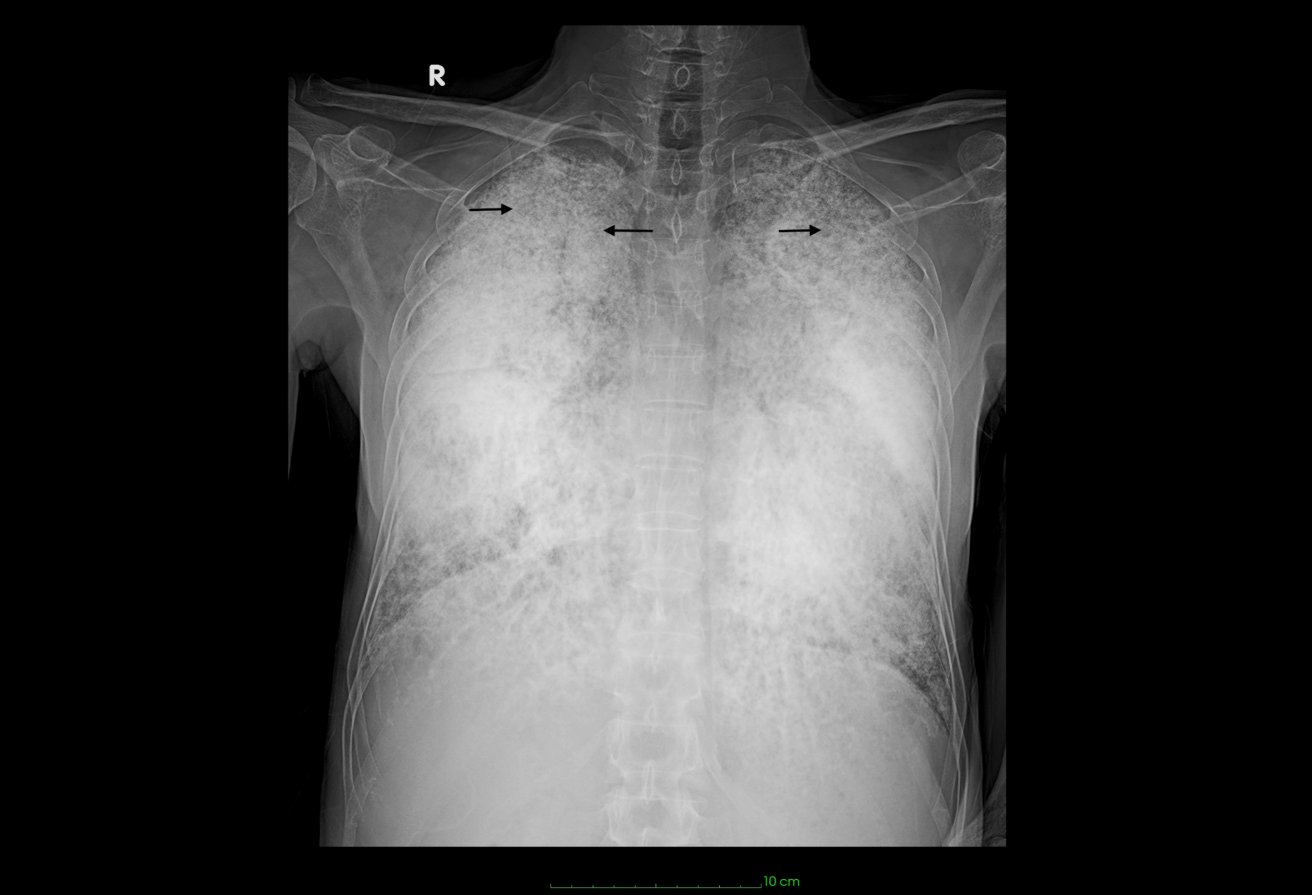
Chest X-ray showing extensive bilateral dense calcific opacities

**Figure 2:**
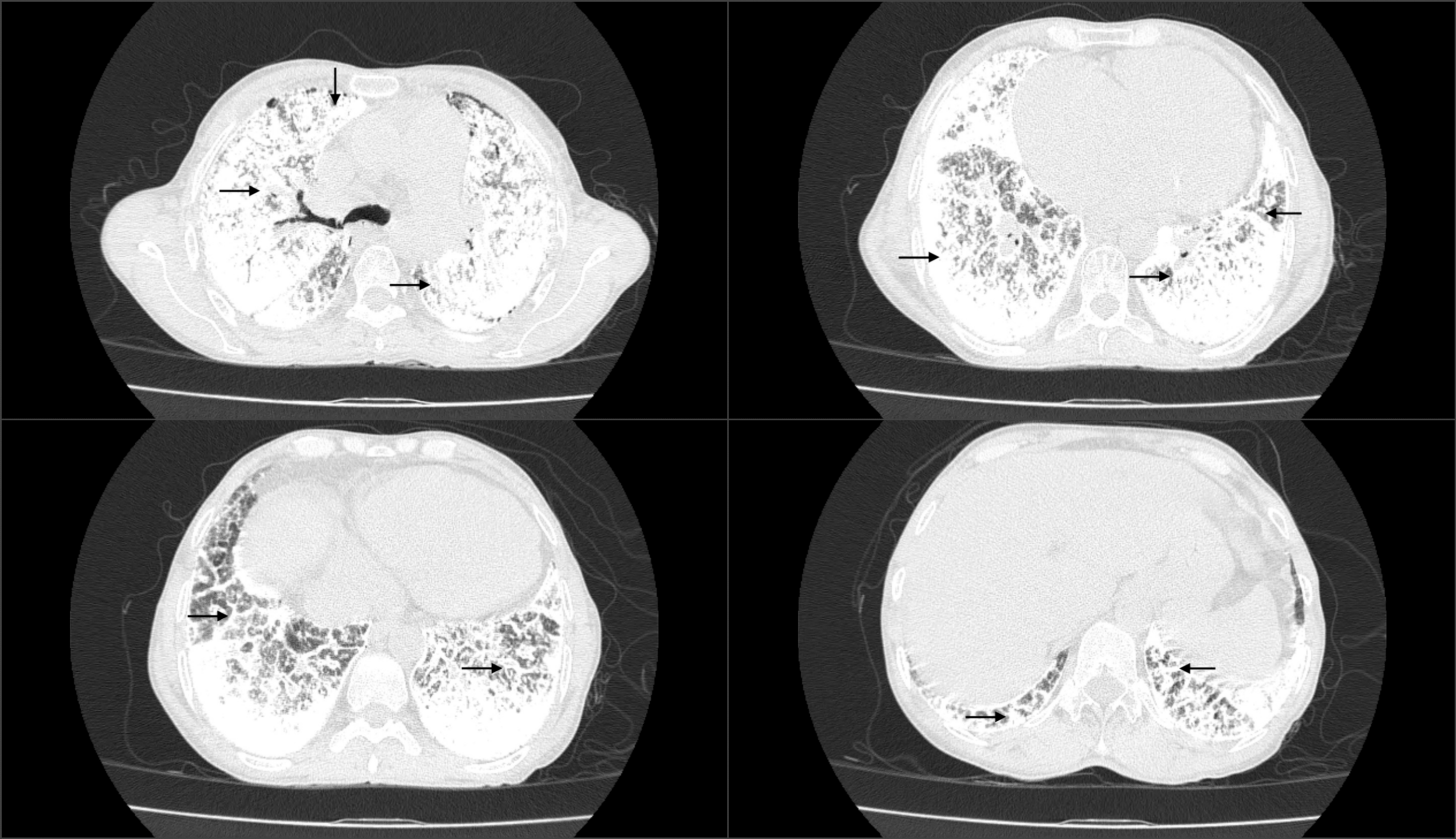
Hight-resolution CT of the chest demonstrating extensive micro-nodular and macro-nodular calcifications - "sandstorm" appearance in both lungs.

Hematological analysis revealed an elevated total leukocyte count of 26.6 × 10³ cells/cubic millimeter, predominantly neutrophils (93.8%), with lymphocytes constituting only 3.8%. Given the presence of fever, productive cough, and neutrophil-predominant leukocytosis, a respiratory infection was initially suspected and appropriately investigated. However, microbiological evaluation, including sputum examination and bronchoalveolar lavage, was negative for acid-fast bacilli, and there was no microbiological evidence of an active bacterial infection. The leukocytosis subsequently improved with intravenous antibiotics and supportive care. Apart from leucocytosis, markedly elevated international normalized ratio (INR) and D-dimer levels, compensated alkalemia with hypoxemia on arterial blood gases were present, while the remaining blood parameters were within normal limits. Details of all investigations, including the reference range, are provided (Table 1). Imaging studies, including a chest X-ray (Figure 1), revealed extensive bilateral calcific dense opacities manifesting as reticulonodular and patchy areas. 2D Echo revealed rheumatic heart disease with moderate mitral stenosis and mild mitral regurgitation, severe tricuspid regurgitation, and severe pulmonary hypertension (107 mmhg), with a preserved left ventricular ejection fraction of 54%. Detailed echocardiographic measurements are provided (Table 2). High-resolution computed tomography (HRCT) of the chest (Figure 2) demonstrated extensive micro-nodular and macro-nodular calcifications resembling sand-like deposits in both lungs, predominantly located along subpleural, peribronchial, and interlobular septae, with associated ground-glass opacification and significant inter/intralobular septal thickening, described as a "crazy paving" pattern, along with areas of patchy consolidation suggestive of the presence of diffuse pulmonary microliths. Bronchoscopy revealed a normal bronchial tree bilaterally without any endobronchial lesions. Bronchoalveolar lavage from the right middle lobe and right lower lobe yielded turbid fluid; microscopy identified calcospherites. Based on these findings, a provisional diagnosis of pulmonary alveolar microlithiasis (PAM) was considered. Genetic analysis identified a disease-causing mutation in the *SLC34A2 *gene, establishing a definitive molecular diagnosis of PAM associated with the observed clinical phenotype. 

**Table 1:** Table summarising laboratory workup on presentation Hb, Hemoglobin; TC, Total Leukocyte Count; PLT, Platelet Count; N, Neutrophils; L, Lymphocytes; E, Eosinophils; M, Monocytes; and B, Basophils. Reticulocyte count assesses immature red blood cells. PT (Prothrombin Time), aPTT (Activated Partial Thromboplastin Time), and INR (International Normalized Ratio) evaluate coagulation status. D-dimer is a marker of fibrin degradation. Renal profile includes BUN (Blood Urea Nitrogen); S. Creatinine, Serum Creatinine; S. Uric Acid, Serum Uric Acid; and electrolytes include Sodium (Na⁺), Potassium (K⁺), Chloride (Cl⁻), Calcium (Ca²⁺), and Magnesium (Mg²⁺). Arterial blood gas (ABG) parameters include pH (acid–base status), pO₂ (partial pressure of oxygen), pCO₂ (partial pressure of carbon dioxide), HCO₃⁻ (bicarbonate), Lac (lactate), and SO₂ (oxygen saturation). The liver profile includes T. Bilirubin (Total Bilirubin), D. Bilirubin (Direct Bilirubin), ALT (Alanine Transaminase), AST (Aspartate Transaminase), ALP (Alkaline Phosphatase), GGT (Gamma-Glutamyl Transferase), Total Protein, and Albumin. In the endocrine profile, RBS stands for Random Blood Sugar, HbA1c for Glycated Hemoglobin (reflecting average glucose control), and TSH for Thyroid Stimulating Hormone. Viral serology results for HIV (Human Immunodeficiency Virus), HBsAg (Hepatitis B surface antigen), and Anti-HCV (antibody to Hepatitis C Virus) were non-reactive.

Parameters	Values	Reference Range
Complete blood count	Hb- 14.7 g/dL	13-17 g/dL
	TC- 26600 cells/cumm	4000 – 11000 cells/cumm
	PLT- 1.69 lakhs/cumm	1.5 – 4.0 lakhs/cumm
Differential count	N- 94%	40 – 80 %
	L- 3.8%	20 – 40 %
	E- 1.2%	1 - 6 %
	M- 0.9%	2 – 10 %
	B- 0.1%	0 – 1 %
Reticulocyte count	1.7%	0.5 – 2.5
Peripheral smear	Normocytic normochromic blood picture	
D-dimer	0.64 microgm/mL	0.00 – 0.5 microgm/mL
Coagulation profile	PT- 41.2 secs	11.51 – 15. 51 secs
	aPTT- 36.3 secs	25.46 – 29.46 secs
	INR- 3.39	0.8 – 1.2
RENAL PROFILE		
Blood urea nitrogen	16.98 mg/dL	7 – 20 mg/dL
Creatinine	0.79 mg/dL	0.6 – 1.2 mg/dL
Uric acid	8.2 mg/dL	3.5 – 7.2 mg/dL
Electrolytes	S. Sodium- 138 mmol/L	136 – 145 mmol/L
	S. Potassium- 3.8 mmol/L	3.5 – 5.1 mmol/L
	S. Chloride- 100 mmol/L	96 – 104 mmol/L
	S. calcium- 8.6 mg/dL	8.5 – 10.5 mg/dL
	S. Magnesium- 2.0 mg/dL	1.5 – 2.4 mg/dL
Arterial blood gases	pH- 7.48	7.34 – 7.45
	pO2- 59 mmHg	75 – 100 mmHg
	pCO2- 43 mmHg	35 – 45 mmHg
	HCO3- 32 mmol/L	22 – 26 mmol/L
	Lac- 1.0 mmol/L	0.36 – 0.75 mmol/L
	SO2- 92%	95- 98%
LIVER PROFILE		
Total bilirubin	1.06 mg/dL	0.1 – 1.2 mg/dL
Direct bilirubin	0.4 mg/dL	0.0 – 0.3 mg/dL
Alanine transaminase	21 U/L	19 – 48 U/L
Aspartate transaminase	17 U/L	19 – 48 U/L
Alkaline phosphatase	137 U/L	55 – 119 U/L
Gamma-glutamyl transferase	69 U/L	0 – 55 U/L
Total protein	6.4 g/dL	4.4 – 7.6 g/dL
Albumin	3.6 g/dL	3.20 – 4. 60 g/dL
ENDOCRINE PROFILE		
	RBS- 112 mg/dL HBA1c – 5.6 %	70 – 110 mg/dL
	TSH - 2.31 microU/mL	0.5 – 8.9
HIV/HBsAg/Anti-HCV	Non- reactive	

**Table 2:** 2D echocardiography report RHD, rheumatic heart disease; MR, mitral regurgitation; AR, aortic regurgitation; TR, tricuspid regurgitation; PH, pulmonary hypertension; PR, pulmonary regurgitation; LV, left ventricle/left ventricular; RV, right ventricle/right ventricular; LVIDd, left ventricular internal diameter in diastole; LVIDs, left ventricular internal diameter in systole; RVDd, right ventricular diameter in diastole; IVSd, interventricular septum in diastole; IVSs, interventricular septum in systole; LA, left atrium/left atrial; RA, right atrium/right atrial; SEC, spontaneous echo contrast; IVC, inferior vena cava; MVOA, mitral valve orifice area; TAPSE, tricuspid annular plane systolic excursion; PA, pulmonary artery; MV GRDT, mitral valve gradient; AF, atrial fibrillation; FVR, fast ventricular rate; GRDT, gradient; EF, ejection fraction

CONCLUSION	Rheumatic Heart Disease (RHD)
	Moderate Mitral Stenosis with Mild+ Mitral Regurgitation (MR
	Trivial Aortic Regurgitation (AR)
	Severe Tricuspid Regurgitation (TR)
	Severe Pulmonary Hypertension (PH)
	Mild Pulmonary Regurgitation (PR)
	Adequate Left Ventricular (LV) Function (Ejection Fraction: 54%)
	Reduced Right Ventricular (RV) Function
	No obvious clot

Measurement	Observed Value (cm)	Normal Range (cm)
LVIDd (LV internal diameter in diastole)	3.60	3.5 – 5.5
LVIDs (LV internal diameter in systole)	1.80	2.4 – 4.2
RVDd (RV diameter in diastole)	3.30	0.7 – 2.1
Aortic Root Diameter	3.00	2.5 – 3.7
LA Dimension	4.60	1.9 – 4.0
IVSd (Interventricular septum diastole)	0.90	0.6 – 1.1
IVSs (Interventricular septum systole)	—	—

Parameter	Findings
Cardiac Position	Normal
Situs	Normal
Systemic Veins	IVC (2.7 cm) Dilated & non-collapsing; Hepatic vein dilated; SEC in IVC
Pulmonary Veins	Normal
Right Atrium	Grossly dilated (6.5 × 7.1 cm); SEC+
Right Ventricle	Grossly dilated
Left Atrium	Dilated (6.3 × 4.1 cm)
Left Ventricle	D-shaped
LA Septum	Thin & bulging towards LA
IV Septum	Normal
Mitral Valve	Thickened & calcified leaflets; submitral fusion;MVOA 1.1 cm²;Dense calcification on lateral commissure
Tricuspid Valve	Lengthy & prolapsing leaflets; TAPSE = 5 mm; Annulus = 38 mm;
Pulmonary Valve	Severe PH: 107mmhg
Aortic Valve	Trileaflets and thickened
Aorta	Normal
Pulmonary Artery	PA & branches dilated
Pericardium	Normal
LV Function (Regional)	Normal
LV Function (Global)	Adequate
Color & Doppler	MV GRDT 15/7 mmHg; MR GRADE I+; AR TRIVIAL; TR GRADE III; PR GRADE I+
Rhythm	Atrial Fibrillation (AF) with Fast Ventricular Rate (FVR)

Management of this patient required a multidisciplinary approach, addressing both the progressive interstitial lung disease and the valvular heart disease. After the diagnosis of PAM was confirmed, the patient and family members were counselled regarding the nature of this rare disease, including its typically slow progression and the lack of any curative medical therapy.

For his pulmonary alveolar microlithiasis, supportive management was initiated. Respiratory support was individualized to improve oxygenation while minimizing adverse cardiac effects. The patient was started on supplemental oxygen at night and during exertion to maintain oxygen saturation above 90%, given his significant pulmonary hypertension and potential for nocturnal desaturation. In addition, long‑term oxygen therapy was instituted to reduce hypoxic vasoconstriction and thereby lower pulmonary artery pressures. Non-invasive ventilation with bilevel positive airway pressure (BIPAP) was applied intermittently during the day and continuously at night, using low pressures with close hemodynamic monitoring to avoid compromising venous return or cardiac output in the setting of underlying rheumatic heart disease. Pulmonary rehabilitation was undertaken, and the patient was advised to receive pneumococcal and influenza vaccinations to prevent respiratory infections that could exacerbate his lung condition.

In terms of his rheumatic heart disease and secondary pulmonary hypertension, we optimized heart failure therapy. Diuretics (furosemide 40 mg daily) were continued to alleviate pulmonary congestion and peripheral oedema. Anticoagulation was initially withheld due to a markedly elevated international normalized ratio (INR) and bleeding risk, then cautiously reintroduced after INR normalised to treat the left lower limb deep vein thrombosis and to prevent further thromboembolic complications related to mitral stenosis. Mitral valve intervention was also evaluated as a potential strategy to reduce the post‑capillary component of pulmonary hypertension. The risks and benefits of balloon mitral valvotomy or surgical intervention were discussed in detail with the patient and his attendants; however, they were unwilling to proceed due to personal and financial reasons, and conservative medical management was therefore continued.

To address the severe pulmonary hypertension, we initiated low‑dose oral sildenafil, a phosphodiesterase‑5 inhibitor, with the aim of ameliorating the precapillary component of pulmonary hypertension, although its efficacy in PAM‑related PH is not well established. While sildenafil is not routinely recommended for pulmonary hypertension due to left heart disease, in this patient, the pulmonary hypertension was of mixed pre‑ and post‑capillary physiology and remained severe (pulmonary artery systolic pressure ~90 mmHg) despite optimal supportive measures and heart failure management. A pulmonology consultation was therefore sought, and a carefully monitored trial of sildenafil was advised on a case‑by‑case basis. Given the patient’s stable systemic blood pressure, sildenafil was initiated and titrated cautiously in accordance with blood pressure, with regular monitoring of systemic blood pressure and oxygenation to detect potential adverse effects. The patient tolerated the medication well, and it was subsequently continued along with advice on a low‑salt diet and daily light exercise as tolerated.

The patient and his family were educated extensively regarding the nature of PAM, as a genetic condition with no definitive medical cure, potentially slowly progressive, with lung transplantation as the only curative option. Given his concomitant cardiac illness, the possibility of a combined heart-lung transplantation in the future was also discussed. However, in view of advanced age and current functional status, medical management was prioritised, and the need for periodically re-evaluating the need for listing for transplantation based on disease progression was emphasised. The patient exhibited a favourable response to treatment and was discharged from the hospital once hemodynamically stable and symptomatically improved, demonstrating significant recovery in respiratory function and overall health status. In the absence of specific guidelines, a pragmatic multidisciplinary follow-up approach was adopted, with initially monthly and later three-monthly reviews focusing on symptom progression, oxygen requirement, functional status, and echocardiographic parameters.

Given the confirmed autosomal recessive inheritance of pulmonary alveolar microlithiasis due to an *SLC34A2 *mutation, the patient and family were counselled about the hereditary nature of the disease. Although no formal genetic or radiological screening of asymptomatic relatives was performed during this admission, family members were advised regarding the option of genetic counselling and targeted evaluation should respiratory symptoms arise, in view of the potential for subclinical or early-stage disease.

## Discussion

The diagnostic approach in this case was particularly challenging due to the presence of extensive miliary calcifications and overlapping cardiopulmonary features. Table 3 outlines the key differential diagnoses considered and the clinical reasoning behind their exclusion. 

**Table 3:** Differential diagnosis of diffuse pulmonary calcific micronodules BAL, bronchoalveolar lavage; AFB, acid-fast bacilli; PAM, pulmonary alveolar microlithiasis; TB, tuberculosis

Differential Diagnosis	Typical Features	Findings in Our Patient	Reason for Exclusion
Miliary Tuberculosis	Diffuse micronodules, usually non-calcified; systemic symptoms (fever, weight loss); positive microbiology	Chronic indolent course; no constitutional symptoms; negative tuberculin test; sputum/BAL negative for AFB; nodules calcified	Miliary TB nodules are typically non-calcified and associated with systemic symptoms
Pulmonary Metastatic Calcification	Seen in chronic renal failure or hyperparathyroidism; upper-lobe predominant calcifications	Normal renal function; normal calcium–phosphate levels; diffuse lung involvement	Absence of metabolic derangement and atypical distribution
Pneumoconiosis (Silicosis)	Upper-lobe predominant nodules; history of occupational exposure; eggshell lymph node calcification	No occupational exposure; no lymph node calcification; diffuse rather than upper-lobe disease	Absence of occupational silica exposure and lack of characteristic upper-lobe-predominant nodules or eggshell lymph node calcification.
Pulmonary Hemosiderosis	Diffuse opacities due to iron deposition; recurrent hemoptysis; hemosiderin-laden macrophages on BAL	No hemoptysis; BAL negative for hemosiderin-laden macrophages	Opacities are calcific, not hemorrhagic
Pulmonary Alveolar Proteinosis	Crazy-paving pattern; proteinaceous alveolar material; low-attenuation opacities	High-attenuation micronodules; BAL showed calcospherites	Imaging and BAL confirmed calcification rather than proteinaceous material
Pulmonary Alveolar Microlithiasis (PAM)	Diffuse calcified micronodules; “sandstorm” appearance; calcospherites on BAL	Classic radiological pattern; calcospherites on BAL; genetic confirmation	Definitive diagnosis

The distinctive radiological pattern, coupled with confirmatory bronchoalveolar lavage and genetic findings, ultimately established pulmonary alveolar microlithiasis as the definitive diagnosis.

Pulmonary alveolar microlithiasis (PAM) is a fascinating, rare lung disease, first described in 1686 and later named as Pulmonary Alveolar Microlithiasis in 1933 by Puhr [4].It is an autosomal recessive condition; mutations in the *SLC34A2 *gene have been identified as the culprit in familial cases [4,5]. *SLC34A2 *encodes a type IIb sodium-phosphate cotransporter on alveolar type II pneumocytes, responsible for clearing phosphate from the alveolar space. Loss-of-function mutations result in phosphate accumulation, which binds with calcium to form insoluble microliths that deposit in the alveoli [4]. Over time, a widespread “sandstorm” appearance envelops the lungs. Histologically, the microliths are concentrically laminated calcium phosphate concretions that gradually enlarge, leading to expansion of alveoli and septal fibrosis [6].

The epidemiology of PAM reveals just over a thousand reported cases worldwide [1,7]. An extensive review by Castellana et al. (2015) documented 1022 cases, noting the highest number of cases in Turkey, Italy, and the USA, though many cases in Asia were underreported in older literature [7]. In India, approximately 86 cases had been reported up to 2018 [1], reflecting the global distribution of this orphan disease. PAM commonly presents in the second to fourth decades of life, but childhood and late adult presentations are known [6]. Our patient’s diagnosis in his 60s is relatively late; it is possible that his disease had been smouldering for decades and only became symptomatic in the setting of his cardiac disease and advancing lung involvement.

Clinically, PAM often has an indolent course in early stages. Patients might be asymptomatic or have only a mild cough. Many cases are detected incidentally on chest X-ray performed for unrelated reasons [6]. As microliths accumulate, lung compliance decreases, and progressive dyspnoea on exertion ensues. Ultimately, chronic hypoxemic respiratory failure and cor pulmonale can develop [6]. Notably, our patient’s respiratory symptoms were initially attributed to his known cardiac condition (mitral stenosis). This case highlights how coexisting pathologies can mask each other. The presence of extensive calcifications on lung imaging in such a context is a red flag that warrants investigation for a second pathology like PAM.

The radiological features of PAM are pathognomonic in advanced disease. A classic chest radiograph shows innumerable tiny sand-like calcified nodules distributed throughout both lungs, often described as a “sandstorm” appearance [8]. The mid and lower lung zones are typically more involved [2]. High-resolution CT (HRCT) provides greater detail: in PAM, HRCT will show innumerable calcified micronodules, usually diffuse. There may be interlobular septal thickening and calcification, giving a calcified “crazy paving” pattern in some cases. Ground-glass opacities can appear due to surrounding alveolitis or microlith accumulation. Subpleural calcifications and thickening can also occur in some cases [8]. In our patient, these characteristic radiological findings were complemented by definitive diagnostic confirmation through bronchoalveolar lavage demonstrating calcospherites and molecular identification of an *SLC34A2 *mutation, and the presence of severe pulmonary hypertension with detailed echocardiographic correlation, thereby strengthening diagnostic certainty in the setting of coexisting rheumatic heart disease.

The concurrence of rheumatic heart disease and severe pulmonary hypertension added complexity to this case. RHD - particularly long-standing mitral stenosis - leads to chronic elevation of left atrial pressure and pulmonary venous pressure, which in turn causes remodelling of pulmonary arterioles and venules (post-capillary pulmonary hypertension). Over time, this can result in severe pulmonary hypertension and right heart failure, even if the primary valve lesion is stable. In our patient, echocardiography confirmed features of both post-capillary PH (due to MS) and pre-capillary PH (indicated by significant right ventricular enlargement out of proportion to the degree of MS, suggesting lung pathology). The findings of pulmonary alveolar microlithiasis in our case appear to be coincidental rather than being pathophysiologically linked to the underlying rheumatic heart disease. However, the pulmonary arterial hypertension in this patient is likely multifactorial, resulting from the combined effects of both conditions. This dual contribution of pulmonary alveolar microlithiasis and rheumatic heart disease to pulmonary hypertension was differentiated through a combination of clinical assessment, imaging, and echocardiographic findings. Clinically, the severity of pulmonary hypertension and right ventricular dysfunction appeared disproportionate to the degree of mitral stenosis alone, suggesting an additional pulmonary vascular or parenchymal component. Echocardiographic assessment demonstrated features consistent with post-capillary pulmonary hypertension attributable to rheumatic mitral valve disease, while high-resolution CT of the chest revealed diffuse pulmonary microliths suggestive of PAM, which was later confirmed by genetic testing. A substantial pre-capillary contribution from pulmonary alveolar microlithiasis was suspected based on the disproportionate severity of pulmonary hypertension relative to valvular pathology, the presence of diffuse calcific lung disease on HRCT, and significant hypoxemia - all supporting a meaningful PAM-related pre-capillary component. In the absence of right-heart catheterization, the pulmonary hypertension was therefore best interpreted as multifactorial. Notably, Sampsonas et al. (2007) described a comparable case of a female with rheumatic valvular disease and suspected PAM, underscoring the rarity of this combination [3]. Our case reinforces that when evaluating a patient with RHD who has unexplained respiratory findings, one must consider concomitant intrinsic lung disease.

Management of PAM is challenging because there is no definitive medical therapy proven to halt or reverse the disease. Lung transplantation remains the only curative option for advanced cases [1,6]. That said, various medical treatments have been attempted with limited success. Corticosteroids have not shown benefit, consistent with the fact that inflammation is minimal in PAM [6]. Bisphosphonates, particularly disodium etidronate, have been reported to inhibit the formation of new pulmonary calcium‑phosphate crystallization and help resolve previously formed calcifications, though data are limited and mixed, and no randomized trials exist [6]. Other therapies under consideration include lavage therapy (whole lung bronchoalveolar lavage to wash out microliths), though microliths are tightly packed and not easily removed [8]. Overall, the evidence supporting both bisphosphonate therapy and lavage remains limited to isolated case reports [9] and small observational studies, with a lack of robust clinical trials. In our patient, these interventions were not pursued due to the unavailability of whole‑lung lavage facilities and cost‑related limitations associated with bisphosphonate therapy [10].

Management, therefore, centres on supportive care: oxygen therapy as needed. There is evidence in the literature that PAM can lead to progressive reduction of capillary beds and interstitial fibrosis, culminating in cor pulmonale [11]. Routine vaccinations, prompt treatment of infections, and minimizing exposure to dust, tobacco, snuff, repetitive lung infections, and cold environments are essential, as these factors may influence both the onset and progression of the disease [12]**.** In our case, management was further tailored to include treatment of pulmonary hypertension. There is no specific guideline for PH in PAM, but general measures for Group 3 PH (PH due to lung disease) were employed, such as long-term oxygen. We cautiously used sildenafil, acknowledging that the evidence for pulmonary vasodilators in lung-disease-associated PH is not robust and must be individualized. Encouragingly, our patient tolerated it well and felt symptomatic relief.

The prognosis of PAM varies widely. Some patients remain stable for many years with only mild symptoms, while others experience progressive respiratory failure over a decade or two. The presence of complications like cor pulmonale typically portends a poorer outcome [6]. Our patient’s prognosis is additionally influenced by his rheumatic heart disease. Uncontrolled PH or heart failure could significantly shorten survival if not managed. On the other hand, if his mitral stenosis is successfully relieved (via intervention) and his PAM remains only slowly progressive, he could potentially have a stable period of several years. Close follow-up is essential.

In summary, our literature review underscores that the coexistence of PAM with RHD and severe PH is exceedingly rare. Each condition individually can cause significant morbidity; together, they present a formidable clinical challenge. Our case highlights both the necessity of vigilance for rare diseases and the value of an integrated treatment strategy. As therapies for PAM are evolving (with ongoing exploration of bisphosphonates and future gene-based treatments), documenting such cases contributes to a better understanding and management of this orphan lung disease.

## Conclusions

This case highlights the rare coexistence of PAM with rheumatic heart disease and severe pulmonary hypertension, underscoring the importance of evaluating for intrinsic lung disease when atypical imaging accompanies dyspnea in known RHD. It reinforces the need for multidisciplinary care and adds to the limited literature on this uncommon association.

In summary, heightened awareness of this rare entity can lead to earlier diagnosis, better symptom management, and improved quality of life in affected patients. Case aggregation may provide further insights into optimal management of PAM and its interaction with other cardiopulmonary conditions. For hypothesis generation, one might speculate whether chronic pulmonary hypertension from mitral stenosis could in any way accelerate microlith deposition by causing repeated episodes of alveolar oedema - an interesting consideration, although current understanding attributes PAM primarily to a genetic metabolic dysfunction. Conversely, it could be hypothesized that the extensive calcifications seen in PAM might contribute to pulmonary vascular stiffening and thereby worsen pulmonary hypertension in patients with underlying rheumatic heart disease. Continued documentation of such cases may help explore these potential interactions.
